# Challenges for the Implementation of Primary Standard Dosimetry in Proton Minibeam Radiation Therapy

**DOI:** 10.3390/cancers16234013

**Published:** 2024-11-29

**Authors:** John Cotterill, Samuel Flynn, Russell Thomas, Anna Subiel, Nigel Lee, Michael Homer, Hugo Palmans, Ludovic De Marzi, Yolanda Prezado, David Shipley, Ana Lourenço

**Affiliations:** 1 Radiotherapy and Radiation Dosimetry, National Physical Laboratory, Teddington TW11 0LW, UK; sam.flynn@npl.co.uk (S.F.); russell.thomas@npl.co.uk (R.T.); anna.subiel@npl.co.uk (A.S.); nigel.lee@npl.co.uk (N.L.); michael.homer@npl.co.uk (M.H.); hugo.palmans@npl.co.uk (H.P.); david.shipley@npl.co.uk (D.S.); ana.lourenco@npl.co.uk (A.L.); 2 Particle Physics Group, School of Physics and Astronomy, University of Birmingham, Edgbaston B15 2TT, UK; 3 Faculty of Engineering and Physical Sciences, University of Surrey, Guildford GU2 7XH, UK; 4 Department of Medical Physics and Biomedical Engineering, University College London, London WC1E 6BT, UK; 5 Medical Physics Group, MedAustron Ion Therapy Center, A-2700 Wiener Neustadt, Austria; 6 Laboratoire d’Imagerie Translationnelle en Oncologie (LITO), Institut Curie, Université Paris-Saclay, Inserm U1288, 91898 Orsay, France; ludovic.demarzi@curie.fr; 7 Radiation Oncology Department, Institut Curie, PSL Research University, 75005 Paris, France; 8 New Approaches in Radiotherapy Lab, Center for Research in Molecular Medicine and Chronic Diseases (CIMUS), Instituto de Investigación Sanitaria de Santiago de Compostela (IDIS), University of Santiago de Compostela, 15706 Santiago de Compostela, A Coruña, Spain; yolanda.prezado@usc.es; 9 Oportunius Program, Galician Agency of Innovation (GAIN), Xunta de Galicia, 15702 Santiago de Compostela, A Coruña, Spain; 10 Institut Curie, Université PSL, CNRS UMR3347, Inserm U1021, Signalisation Radiobiologie et Cancer, 91400 Orsay, France; 11 Université Paris-Saclay, CNRS UMR3347, Inserm U1021, Signalisation Radiobiologie et Cancer, 91400 Orsay, France

**Keywords:** dosimetry, calorimetry, proton, minibeams, SFRT, pMBRT

## Abstract

**Background/Objectives**: Spatial fractionation of proton fields as sub-millimeter beamlets to treat cancer has shown better sparing of healthy tissue whilst maintaining the same tumor control. It is critical to ensure primary standard dosimetry is accurate and ready to support the modality’s clinical implementation. **Methods**: This work provided a proof-of-concept, using the National Physical Laboratory’s Primary Standard Proton Calorimeter (PSPC) to measure average absorbed dose-to-water in a pMBRT field. A 100 MeV mono-energetic field and a 2 cm wide SOBP were produced with a spot-scanned proton beam incident on a collimator comprising 15 slits of 400 µm width, each 5 cm long and separated by a center-to-center distance of 4 mm. **Results**: The results showed the uncertainty on the absorbed dose-to-water in the mono-energetic beam was dominated by contributions of 1.4% and 1.1% (k = 1) for the NPL PSPC and PTW Roos chambers, respectively, originating from the achievable positioning accuracy of the devices. In comparison, the uncertainty due to positioning in the SOBP for both the NPL PSPC and PTW Roos chambers were 0.4%. **Conclusions**: These results highlight that it may be more accurate and reliable to perform reference dosimetry measuring the Dose-Area Product or in an SOBP for spatially fractionated fields.

## Simple Summary

Proton minibeam radiation therapy (pMBRT) is a technique using spatial fractionation of a proton field to treat cancer. Evidence suggests there is improved sparing of healthy tissue when spatially fractionating the beam without any loss of tumor control. Development of this technique could therefore improve the clinical outcome for patients. Primary standard dosimetry will be required for safe implementation in the clinic. The spatial fractionation of the field, however, makes the dosimetry challenging, as small positioning uncertainties for devices used for dosimetry can lead to large differences in the measured dose. This work examined the current feasibility of operating a primary standard-level dosimetry device in an experimental pMBRT field, and suggests dosimetric improvements necessary to assist the adoption of the technique in the clinic.

## 1. Introduction

External beam radiotherapy contributes to approximately 50% of cancer treatments, either solely or in combination with other techniques [1,2,3]. The radiation delivery is optimized to ensure a sufficient dose is delivered to the tumor for the required tumor control, whilst minimizing the dose delivered to surrounding healthy tissue. Different particle types each have their own particular advantageous characteristics which allows for particle selection based on the clinical benefit for different cancers. For example, the distinct end of range seen with protons allows for strategic positioning of the particles to limit the dose delivered to the healthy tissue beyond the tumor volume. This allows for targeting of tumors in close proximity to critical structures in the body where the dose must be minimized, such as in the head and neck.

Not only does the particle type impact the efficacy of the treatment, but also the method through which it is delivered. Advanced treatment methods are continually being developed and adopted clinically, such as IMRT [4,5,6], Ultra-High Dose Rate (UHDR)/FLASH radiotherapy [7,8,9,10,11,12,13,14], and MR-guided radiotherapy [15,16,17,18,19,20]. Another promising modality of proton therapy, termed proton minibeam radiation therapy (pMBRT), is under development which delivers the proton beam in a spatially fractionated manner [21,22,23,24,25]. This spatial fractionation consists of multiple small beamlets, each typically 400–1000 µm in width. Beam delivery in this way has been shown to reduce the toxicity observed in healthy tissues on entry to the patient in comparison to typically utilized homogeneous beams [26,27,28,29]. However, the multiple Coulomb scattering occurring to the protons in each beamlet through the patient can be used to achieve a homogeneous dose distribution to the tumor more comparable with conventional proton beam therapy. This technique therefore allows the targeting of deep-seated tumors whilst improving skin sparing and upstream healthy-tissue sparing.

Accurate dosimetry traceable back to primary standard absolute dosimetry is critical for the safe implementation of radiotherapy in the clinic. An International Code of Practice (TRS-398 Rev. 1 [30]) published by the International Atomic Energy Agency (IAEA) recommends standard practices to maintain accuracy and consistency between facilities in broad-beam conditions. These practices work well for established radiotherapy modalities; however, they have been found to be inadequate for new modalities coming to the fore such as FLASH radiotherapy [31,32,33,34], MR-guided radiotherapy [35,36,37,38] and minibeam radiotherapy. It is therefore important for National Measurement Institutes (NMIs) to develop devices and protocols to perform this dosimetry as these new modalities are adopted.

The small size of the beamlets used in pMBRT is not addressed by the reference conditions in IAEA TRS-398. Recommendations for the dosimetry in small fields are made in IAEA TRS-483 [39], but the field sizes addressed have lower limits of 4 mm, and are thus still not of comparable size to those used in pMBRT. Alternative methods for performing dosimetry in pMBRT are therefore required, with some techniques having been proposed [40,41]. So far, no primary standard instrument has been utilized in these fields, yet is a critical part of the traceability chain to ensure accuracy and consistency.

There is an additional challenge for the dosimetry in pMBRT as there has been no consensus reached on the accepted dose metric as the critical descriptor for these fields, or whether a combination of these metrics is required. Various metrics have been proposed, such as average (mean) dose, Equivalent Uniform Dose (EUD), peak dose, valley dose, and peak-to-valley dose ratio (PVDR) [25,42,43,44,45,46,47]. This work discusses the experimental investigation of the current feasibility of performing dosimetry in minibeam fields with the National Physical Laboratory’s (NPL) Primary Standard Proton Calorimeter (PSPC) at the Institut Curie—Centre de Protonthérapie d’Orsay, France. The utilization of the NPL PSPC for this work is in line with the measure of average (mean) dose, though this does not suggest that this is the critical metric.

The aim of this work was to examine the feasibility of using the NPL PSPC in minibeams, as well as identify and quantify the challenges of performing dosimetry with a primary standard device in such complex fields. Both a 100 MeV mono-energetic field and an SOBP field were examined to compare their suitability as conditions for reference dosimetry. The impact of detector-positioning uncertainties, as well as collimator tilt and non-uniformities in the field, were examined to determine the impact on the dose measurements. Suitable uncertainty estimates for various sources were calculated and compared to determine the most impactful changes necessary for improving accuracy in the dosimetry. The work of this study consequently supports the development of effective dosimetry protocols to enable the safe implementation of the modality in the clinic.

## 2. Materials and Methods

### 2.1. NPL Primary Standard Proton Calorimeter

The NPL PSPC constitutes a 2 mm thick graphite core with a diameter of 16 mm. The core is contained inside two graphite jackets and a mantle, each separated by a 0.75 mm wide gap maintained under high-quality vacuum. These vacuum gaps help to reduce the heat flow between components of the calorimeter and enhance the ability to measure the temperature rise solely induced by the radiation. Each component of the calorimeter is embedded with thermistors for temperature monitoring and electrical heating. A more detailed description of the NPL PSPC configuration is given in Lourenço et al. [34].

The calorimeter was operated in both quasi-adiabatic (QA) and active isothermal modes. In quasi-adiabatic mode, the temperature of the outermost jacket is electronically controlled using thermistors as heating elements, whilst the temperature of the core is allowed to drift. This creates a stable thermal environment for the core, providing shielding from local temperature fluctuations. The radiation-induced temperature rise, after accounting for drift during the irradiation, can be measured and used to determine the dose delivered to the core.

In active isothermal mode, thermistors are used as heating elements to maintain the core at a fixed temperature above ambient. The electrical power required to maintain the temperature at the required level reduces as radiation is delivered, due to radiation-induced heating. The reduction in electrical power integrated over the radiation delivery time can then be used to calculate the dose. Operating the calorimeter in both modes, and taking the mean value, increases the robustness of the dose measurement using the device.

The absorbed dose-to-water averaged over the sensitive area of the calorimeter is derived from a measurement in the NPL PSPC, according to the equation

$$D_{w}=D_{core}^{m}\cdot \frac{D_{w}^{MC}}{D_{g}^{MC}}\cdot k_{imp}k_{gap}k_{vert.}\;$$

$D_{core}^{m}$ is the measured dose-to-core, calculated as the product of the radiation-induced temperature rise, ∆T, and the specific heat capacity of the core, c, in QA mode or from the integrated electrical power reduction divided by the core mass in active isothermal mode. $D_{w}^{MC}/D_{g}^{MC}$ is the Monte Carlo (MC)-derived dose conversion factor, defined as the product of the water-to-graphite mass stopping power ratio, $s_{w,g}$, and the fluence correction factor, $k_{fl}$ [48], and converts the dose-to-graphite to dose-to-water. The $k_{imp}$ correction factor corrects for the non-graphite constituents of the calorimeter, such as core thermistors and impurities within the graphite. The $k_{gap}$ correction factor accounts for the presence of the vacuum gaps between the core and surrounding jackets. A $k_{vert}$ correction factor was required (due to space limitations of the experimental set-up) to account for the difference in sampled area of the beam due to the vertical position of the calorimeter core with respect to the beam central axis (see Section 2.2).

### 2.2. Experimental Set-Up

**Figure 1:**
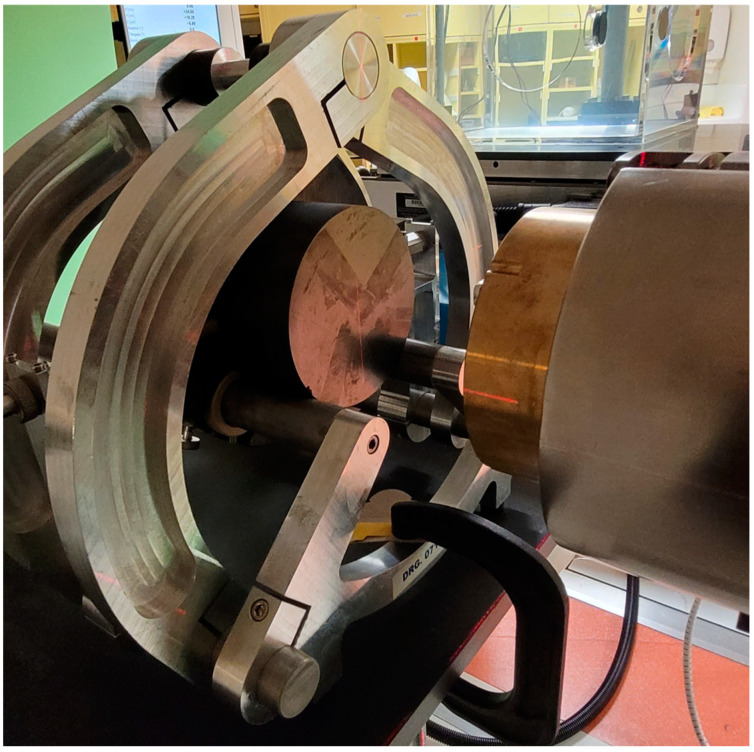
Experimental set-up of the NPL Primary Standard Proton Calorimeter (PSPC) downstream from the brass minibeam collimator.

**Figure 2:**
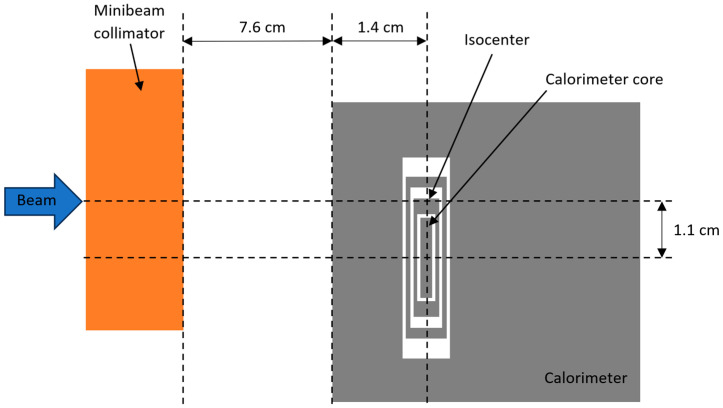
Schematic diagram (not to scale) of the set-up for a 100 MeV mono-energetic spot-scanned proton beam with the NPL Primary Standard Proton Calorimeter (PSPC). The NPL PSPC was positioned 11 mm below the center of the field.

Figure 1 shows the experimental set-up of the NPL PSPC at the facility. Figure 2 shows a schematic representation of the set-up of the calorimeter when operated with a 100 MeV mono-energetic spot-scanned proton beam.

A proton pencil beam was scanned over the upstream surface of a specifically designed collimator to produce the planar minibeam distribution, as described in De Marzi et al. [49]. The collimator was made of 6.5 cm thick brass and mounted on the end of the beam nozzle. It comprised 15 slits which were 400 µm wide and 5 cm long, each separated by 4 mm (center-to-center). Each slit was angled to account for the divergence of the scanned pencil beam. A detailed discussion of the robustness of set-up parameters for a similar configuration is described by Ortiz et al. [50]. Two proton beam configurations were investigated:

1. A 100 MeV mono-energetic spot-scanned beam;
2. A 123 MeV Spread-Out Bragg Peak (SOBP) with an extension in depth of 2 cm centered at a depth of 10 cm in water.

The irradiated field size was 7.2 × 7.2 cm^2^ and consisted of 625 regularly spaced—3 mm—scanned pencil beams on a Cartesian grid across the collimator. Details of the beamline and spot characteristics are described in detail by De Marzi et al. [51]. A larger size of irradiated field than the maximum width between the furthest slits, and a spot spacing less than the size of the spots, was used to ensure dose uniformity across the collimator. The isocenter was located 9.0 cm downstream from the back face of the collimator. Independent measurements were performed with the NPL PSPC and three PTW Roos (Type 34001) chambers at a reference water-equivalent depth (WED) of 2.0 cm for the mono-energetic beam. The WED of 2.0 cm was achieved with the PTW Roos chambers by positioning them in a water phantom whilst accounting for the water-equivalent thickness of the phantom wall and chamber entrance window. The water phantom used was approximately 30 × 30 × 30 cm^3^ to ensure there were no scattering effects from the boundaries of the phantom. Graphite buildup plates were placed in front of the NPL PSPC to achieve the 2.0 cm WED of the center of the core, as the calorimeter is not submersible. The thickness of the graphite buildup plates were determined from previous experimental-range measurements in water, both with and without the graphite positioned in front of a water phantom. Dose-to-water was determined from measurements with the PTW Roos chambers according to the code of practice described in IAEA TRS-398 Rev. 1 [30]. A $k_{prof}$ correction factor was required to correct for the difference in the dose measured resulting from the differing size of the sensitive region of the PTW Roos chambers compared to the calorimeter core.

A 7.0 cm airgap between the back face of the collimator and the buildup material was used for the measurements in the SOBP in water to match that used with the mono-energetic beam. The reference point of measurement using the NPL PSPC and the PTW Roos chambers was in the center of the SOBP at 10.0 cm WED. The calorimeter was similarly not submersed in water, so the airgap and buildup thickness of graphite was scaled to achieve the same physical distance between the collimator and reference point of measurement as the PTW Roos chamber measurements in water.

Space limitations when performing the experiment meant the center of the NPL PSPC core had to be positioned 11 mm vertically below isocenter, as indicated in Figure 2, when operated with the mono-energetic beam. This was deemed acceptable for this proof-of-concept study, given the 5 cm vertical extension of the slits. Measurements were performed with the PTW Roos chambers positioned at both isocenter and 11 mm below isocenter for comparison with the NPL PSPC measurements. The calorimeter measurements with the SOBP were performed in the center of the field without this vertical displacement.

Further measurements were performed with EBT3 and EBTXD radiochromic films at the phantom surface and at the measurement depths of 2.0 cm WED for the 100 MeV mono-energetic beam, and 10.0 cm WED in the SOBP. These measurements were used for the optimization of Monte Carlo-based simulations, as well as the calculation of the required correction factors. The radiochromic films were calibrated against films irradiated in a 100 MeV mono-energetic, 10 × 10 cm^2^ field without use of the minibeam collimator. The calibration films were positioned at 2.0 cm WED with 10.0 cm backscatter using water equivalent plastic, and originated from the same film batch as the measurement films. For the EBT3 films, a 12-point dose scale was used (0, 0.25, 0.5, 1, 2, 3, 4, 5, 6, 8, 10, and 15 Gy). For the EBTXD, a 16-point calibration scale was used (0, 0.25, 0.5, 1, 2, 3, 4, 5, 7.5, 10, 15, 20, 30, 45, 60, and 80 Gy). All films were scanned at 600 dpi at NPL using an Epson Expression 10000XL flatbed scanner. An average of five scans for each film piece (both measurement and calibration) was performed to reduce random noise. Due to beam access limitations, the measurement and calibration radiation exposures were not performed on the same week. To account for this, a period of one month following exposure was left before scanning, to ensure that all films had self-developed by the same amount. Handling of all films was performed using powder-free nitrile gloves to minimize organic contamination, with all films scanned in the same orientation. The EBT3 radiochromic film was used for analysis, whilst the EBTXD was used for independent validation of the setup.

### 2.3. Dose Uncertainty Due to Positioning

Small offsets in the detector position from the target position in the field would be expected, due to the precision in maneuvering the device and the accuracy of the laser alignment system. The spatial fractionation of the beam meant that a small horizontal offset in the sensitive region of a detector could have a significant impact on the measured dose from the different sampled field distribution. The extent of this impact was quantified for both beam configurations by calculating the Dose-Area Product (DAP) per unit area over the sensitive area of the NPL PSPC and PTW Roos chamber from an experimentally measured two-dimensional (2D) profile using EBT3 and EBTXD radiochromic film at the measurement point. The calculation of the DAP per unit area was repeated for horizontal offsets between −3 mm and +3 mm in 0.5 mm increments from the center of the beam, at a fixed vertical displacement of −11 mm. The −11 mm vertical displacement was to coincide with the experimental set-up of the calorimeter in the mono-energetic field. The DAP per unit area for each horizontal offset was then normalized to the DAP per unit area at 0 mm horizontal offset, to give the relative difference from that expected with central horizontal alignment. Although PTW Advanced Markus and PTW Bragg Peak chambers were not used experimentally, they have been considered for this analysis for the purpose of examining the impact of different sized detector regions.

A similar consideration was made for the achievable vertical positioning. The DAP per unit area was calculated for vertical offsets from the measurement position of −9 mm to −13 mm in 0.5 mm increments, at a fixed horizontal displacement of 0 mm. The DAP per unit area for each offset was normalized to the DAP per unit area at −11 mm vertical offset to give the relative difference from that expected with perfect vertical alignment at −11 mm displacement.

This uncertainty due to positioning was examined to estimate an adequate Type B uncertainty contribution on the experimental dose measurements. This uncertainty also has an impact on the calculation of the $k_{vert}$ and $k_{prof}$ correction factors, as these correspond to the positioning and sampling regions of the detectors, respectively. The analysis was repeated for both the mono-energetic and SOBP fields.

Although calibrated, no correction was applied for quenching of the film. Any impact due to quenching would be more dominant and complex in the SOBP field in comparison to the mono-energetic field, due to being near the fall-off region of multiple Bragg peaks where there was higher LET. However, any quenching would have had negligible impact on the results, due to the normalization relative to the DAP per unit area at the target measurement position in the same film. A quenching correction would have needed to be applied only if any comparison between the measured doses on the film were to be compared with the calorimeter and ionization chamber dose measurements directly.

### 2.4. Monte Carlo Model

A model of the beam and the collimator was used to calculate the Monte Carlo-derived correction factors using TOPAS (v3.6.1), based on Geant4 v10.6.p03 [52,53]. Simulations were performed with the default reference physics lists suitable for applications in proton therapy, which included g4em-standard_opt4, g4h-phy_QGSP_BIC_HP, g4decay, g4ion-binarycascade, g4h-elastic_HP, and g4stopping [54].

An attempt was made to optimize the collimator tilt angle relative to the incident beam in the model based on experimentally measured 2D profiles using radiochromic film to reduce the Type B uncertainties associated with the modelling, as small tilt angles of the collimator have been shown to significantly impact the field distribution [50]. Challenges with this optimization meant a larger Type B uncertainty was applied, whilst assuming no collimator tilt. This optimization is discussed in Section 3.2.

The model of the NPL PSPC was built using the dimensional and materials metrology performed at NPL of the constituents of the physical device (e.g., graphite impurities and thermistor materials). Water and graphite materials were defined with ionization potentials of 78 eV and 81 eV, respectively, in accordance with the recommendations in the *International Commission on Radiation Units and Measurements (ICRU) Report 90* [55].

### 2.5. Monte Carlo-Derived Correction Factors

#### 2.5.1. $k_{imp}$ and $k_{gap}$

The $k_{imp}$ and $k_{gap}$ correction factors were calculated by modelling the experimental set-up in three different configurations:

1. Full geometry: containing non-graphite materials such as thermistors, and impurities within the graphite.
2. Pure graphite geometry: non-graphite materials and impurities are substituted for pure graphite. All vacuum gaps remain present.
3. Compensated geometry: vacuum gaps are shifted downstream and filled with pure graphite to make the material homogeneous. This maintains the same buildup depth in front of the core.

The $k_{imp}$ correction factor was then calculated as the ratio of the dose scored in the core volumes of configurations 2 and 1. This factor converted the dose-to-core containing impurities to dose-to-core in pure graphite. The $k_{gap}$ correction factor was calculated as the ratio of the dose scored in the core volumes of configurations 3 and 2. This factor converted the dose-to-core in pure graphite to dose-to-core in homogeneous pure graphite.

#### 2.5.2. Dose Conversion Factor

The dose conversion factor was calculated following the approach described by Palmans et al. [48], to convert the dose-to-graphite to dose-to-water. This involved simulating large volumes of both homogeneous pure graphite and water with cylindrical scoring regions of 16 mm diameter to match that of the NPL PSPC core. The dose as a function of depth was calculated in both materials to give Percentage Depth Dose (PDD) curves for the 100 MeV mono-energetic beam. The PDD for graphite was then scaled such that the range at which the maximum dose decreased to 80%, $r_{80}$, matched that in water, to give the dose at a water equivalent depth. The ratio of the dose scored at 2.0 cm between the PDD for water and the scaled PDD for graphite was taken as the dose conversion factor, $D_{w}^{MC}/D_{g}^{MC}$, for the 100 MeV mono-energetic beam. This procedure was repeated for the simulated SOBP, with the ratio between the dose scored at 10.0 cm between the PDD for water and the scaled PDD for graphite being taken as the dose conversion factor for the SOBP measurements.

### 2.6. Film-Derived Correction Factors

#### 2.6.1. $k_{vert}$

Non-uniformity, along individual beamlets in the minibeam distribution, was observed from the radiochromic film measurements. This inhomogeneity meant that the vertical position of measurement would impact the measured dose. It was therefore appropriate to apply a vertical correction factor, $k_{vert}$, to correct for the difference in the sampled beam distribution between vertical positions of 0 mm and −11 mm from the center of the field. This correction factor was calculated as the mean value of the ratio between the DAP per unit area in the sensitive region of the detector at −11 mm and 0 mm vertical offset for 6 repeated radiochromic film measurements (3 EBT3 and 3 EBTXD) at 2 cm WED. The standard deviation was taken as the Type A uncertainty. Applying this correction allowed the measurements at 0 mm vertical offset to be compared with those at −11 mm vertical offset. Separate values of the $k_{vert}$ correction factor were calculated for the PTW Roos chambers ($k_{vert}(Roos)$) and the NPL PSPC ($k_{vert}(PSPC)$), to correct their respective measurements. The same analysis was performed with 2 EBT3 radiochromic film measurements in the SOBP field at 10 cm WED.

#### 2.6.2. $k_{prof}$

The profile correction was used to correct for the difference in the dose measured resulting from the differing size of the sensitive regions of the PTW Roos chamber and NPL PSPC. The DAP per unit area was calculated over the sensitive area of the PTW Roos (7.8 mm radius) and NPL PSPC (8.0 mm radius) at −11 mm vertical offset for 6 repeated radiochromic film measurements (3 EBT3 and 3 EBTXD) at 2.0 cm WED. The ratio between the DAP per unit area in the NPL PSPC and the PTW Roos was calculated for each film, and the mean value was taken as $k_{prof}$. The standard deviation of the values was taken as the Type A uncertainty. The same analysis was performed with 2 EBT3 radiochromic film measurements for the $k_{prof}$ correction factor in the SOBP.

### 2.7. NPL PSPC Measurement

Measurements were performed in both quasi-adiabatic mode and active isothermal mode using the NPL PSPC, as outlined in Section 2.1. Further detail on these techniques is given in Lourenço et al. [56].

## 3. Results

### 3.1. Dose Uncertainty Due to Positioning

**Figure 3:**
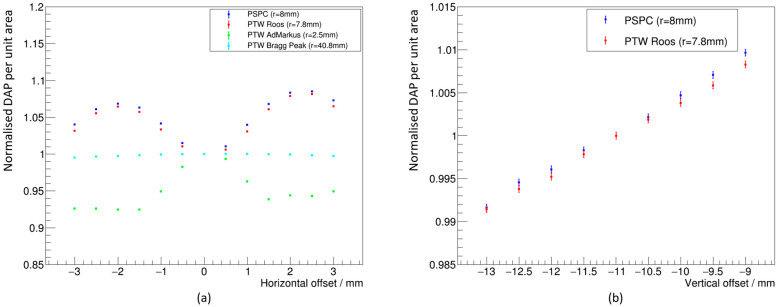
Variation in the Dose-Area Product (DAP) per unit area for different sized sensitive regions in the 100 MeV mono-energetic field, at different (**a**) horizontal offsets and (**b**) vertical offsets. Results were normalized to the DAP per unit area with 0 mm and −11 mm offsets, respectively.

Figure 3a shows the DAP per unit area for different horizontal offsets, at a fixed vertical offset of −11 mm, normalized to the DAP per unit area at 0 mm horizontal offset in the 100 MeV mono-energetic field at 2.0 cm WED. Figure 3b shows the DAP per unit area for different vertical offsets, at a fixed horizontal offset of 0 mm, normalized to the DAP per unit area at −11 mm vertical offset in the same beam.

**Figure 4:**
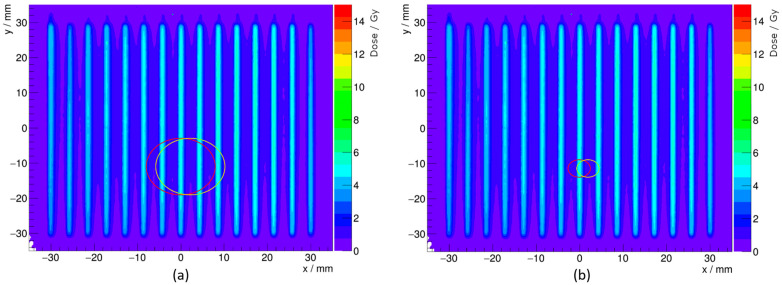
Position of the sensitive regions overlaid on top of processed radiochromic film for the (**a**) NPL PSPC and a (**b**) PTW Advanced Markus on the 100 MeV mono-energetic minibeam field with 0 mm (red) and 2 mm (yellow) horizontal offsets, both at −11 mm vertical displacement.

Figure 3a indicates that a 2 mm horizontal offset to the NPL PSPC or PTW Roos chamber would result in an increase in the dose measured by approximately 8% compared to the dose if it were centrally aligned. In comparison, a 2 mm horizontal offset to a PTW Advanced Markus chamber in the same field would have resulted in an approximate 8% reduction in dose compared to central alignment. The opposing direction in the change of dose is a result of the interplay between the minibeam configuration and the size of the detectors’ sensitive region. The 2.5 mm radius of the sensitive region of the PTW Advanced Markus is small enough to enable it to mostly fit between two neighboring beamlets, consequently losing dose. In comparison, the sensitive region of the NPL PSPC or PTW Roos results in a significant proportion of an additional beamlet being detected, and so increases the dose measured. The position of the sensitive regions of the NPL PSPC and PTW Advanced Markus on the minibeam field are shown in Figure 4 for a 2 mm horizontal offset. A sensitive region with a radius of 40.8 mm was also considered to represent a PTW Bragg Peak chamber. In such a case, there is a reduction in dose measured by <1% for a 2 mm offset because the minibeam field is almost entirely encapsulated, so the offset has little impact on the dose measured.

The near-linear relation shown in Figure 3b highlights the inhomogeneity along individual beamlets. This inhomogeneity is the reason for the $k_{vert}$ correction factor, which accounts for the difference in the dose measurement as a function of vertical position in the field.

The relationships shown in Figure 3 were used to calculate a Type B uncertainty on the dose measurement resulting from the uncertainty on the detector positioning. It was assumed that the detectors could be positioned within ±1 mm of the coordinates (0, −11) mm on the minibeam lateral dose distribution with 95% confidence according to a Gaussian distribution (mean = (0, −11) mm, σ = 0.5 mm). The distributions of the normalized DAP per unit area for both horizontal and vertical offsets shown in Figure 3 for the NPL PSPC and PTW Roos chamber were sampled according to this Gaussian distribution. The standard deviation of the sampled values from the distributions based on the horizontal and vertical offsets were taken as the Type B uncertainties due to horizontal and vertical positioning uncertainties, respectively. These uncertainties were added in quadrature to calculate an overall uncertainty on the dose measurement based on 2D positioning uncertainty of the detectors. The resulting overall Type B uncertainties on the dose measurements at (0, −11) mm in the mono-energetic field for the NPL PSPC and PTW Roos chamber due to 2D positioning uncertainty were 1.4% and 1.1%, respectively.

**Figure 5:**
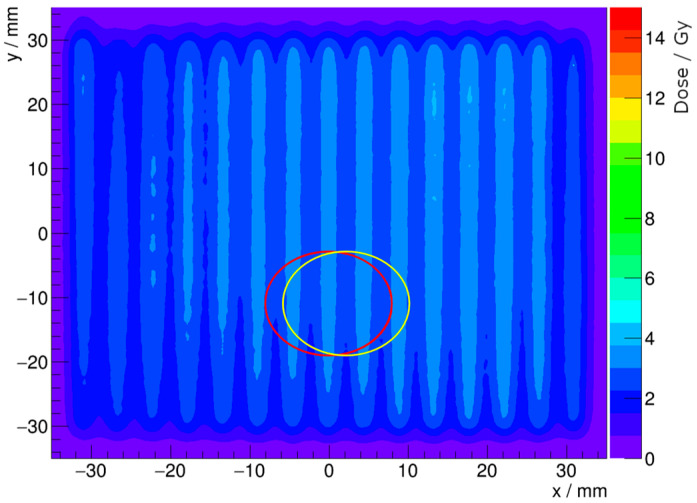
Position of the sensitive region for the NPL PSPC on the SOBP field with a 0 mm (red) and 2 mm (yellow) horizontal offset, both at −11 mm vertical displacement, overlaid on top of processed radiochromic film.

The same analysis was performed for the SOBP. Figure 5 shows the position of the sensitive regions of the NPL PSPC with 0 mm and 2 mm horizontal offset from the horizontal center of the field. The difference in the dose measured at a 2 mm horizontal offset compared with central alignment for the SOBP was <3%, smaller than the 8% difference in the mono-energetic field. The resulting overall Type B uncertainties on the dose measurements at (0, −11) mm in the SOBP field for the NPL PSPC and PTW Roos chamber due to 2D positioning uncertainty were both 0.4%. This significantly smaller uncertainty compared to the mono-energetic beam is because the scatter of the protons gives a more uniform field (beamlets are still distinct, but to a lesser extent) at the point of measurement at 10.0 cm WED in comparison to the 2.0 cm WED in the mono-energetic field. The position uncertainty of the sensitive region, therefore, has a smaller contribution to the Type B uncertainty on the correction factors, since there is a reduced change in the sampled dose distribution from a small offset.

### 3.2. Collimator Tilt Optimization

**Figure 6:**
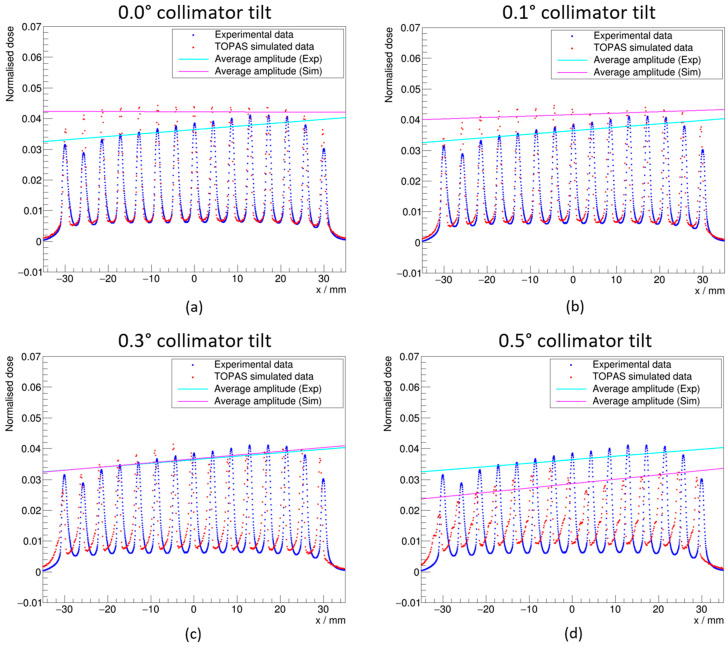
Simulated dose distributions at 2.0 cm WED for collimator tilt angles of (**a**) 0.0°; (**b**) 0.1°; (**c**) 0.3°; and (**d**) 0.5°, normalized to the total dose across the field. Comparison is made with the experimental data to best quantify the unknown tilt. (Exp) and (Sim) refer to experiment and simulation, respectively.

An attempt to optimize the collimator tilt in simulation was performed to achieve the most accurate representation of the incident dose distribution during the experimental campaign. The simulated 2D dose distribution at a depth of 1.2 cm in graphite (2.0 cm WED) was compared with the experimentally measured equivalent set-up at collimator tilt angles of −0.5° to +0.5° in 0.1° increments. The resultant distributions for angles of 0.0°, 0.1°, 0.3° and 0.5° are shown in Figure 6.

Figure 6 indicates that a collimator tilt angle of 0.3° achieves an average peak magnitude and gradient of the peak magnitude that matches that measured experimentally. However, the asymmetry within an individual peak was observed to trend in the opposite direction (most apparent in Figure 6d). The trend observed in the simulation follows that previously reported by Ortiz et al. [50], which indicated that there was an effect within the experimental beam set-up which reversed the trend. Consequently, it was not possible to achieve a collimator tilt angle which appropriately represented the beam observed experimentally. Calculation of the Monte Carlo-derived correction factors was therefore carried out with a 0.0° tilt to the collimator, and a larger Type B uncertainty was applied.

### 3.3. Monte Carlo-Derived Correction Factors

The values of the Monte Carlo-derived correction factors $k_{imp}$, $k_{gap}$, and $D_{w}^{MC}/D_{g}^{MC}$, for both mono-energetic and SOBP fields are shown in Table 1. The $k_{imp}$ and $k_{gap}$ correction factors are comparably close to unity with previously calculated values for the NPL PSPC [22] and with graphite calorimeters of differing design [57] in proton fields. Though not directly comparable due to differences in the incident beam properties and experimental set-ups, the small difference between the values is indicative of the fact that a minibeam field has little impact on their magnitude in comparison to broad homogeneous proton fields. The dose conversion factor value of 1.1164 was larger than previously reported values using the NPL PSPC or similar graphite calorimeters. However, this is expected, due to the lower proton energy of 100 MeV in this case, compared to the nominal 250 MeV proton beam reported previously [34]. This is because the dose conversion factor incorporates a fluence correction factor, which accounts for the difference in the fluence between water and graphite at water-equivalent depths. This difference arises from variations in non-elastic nuclear-interaction cross sections and secondary particle-production cross sections between these materials. It depends on both initial energy, depth of measurement and scattering conditions, leading to a differing magnitude at 100 MeV compared to 250 MeV.

Type B uncertainties were estimated based on simulations performed with realistic collimator tilts, horizontal offsets, and ionization potentials of water and graphite. The correction factors were calculated with collimator tilts between 0.0° and 0.5° in 0.1° degree increments, and horizontal offsets of −2 mm, 0 mm, and 2 mm. The correction factors were also calculated for ionization potentials of water between 74 eV and 82 eV in 2 eV increments (nominal of 78 eV), and graphite between 77.4 eV and 84.6 eV in 1.8 eV increments (nominal of 81 eV). These increments were used for the ionization potentials, as they represent the standard uncertainty on the nominal value in the ICRU Report 90 [55]. The maximum deviation of these correction factors from their nominal value was considered as the expanded uncertainty (k = 2). The uncertainties were then combined in quadrature. Differences between nuclear models were not considered when estimating the uncertainty, as the measurements performed were not at primary standard level. This study was instead focused on identifying the main sources of uncertainty and challenges of performing measurements with a primary standard device. Differences between nuclear models should be considered when performing complete primary standard dosimetry and compilation of a full uncertainty budget.

### 3.4. Film-Derived Correction Factors

The $k_{vert}$ and $k_{prof}$ correction factors are shown in Table 2. The >2% deviation from unity of the $k_{vert}$ correction factor for both the mono-energetic and SOBP fields demonstrates the impact of the inhomogeneity along individual beamlets, necessitating the vertical correction to the measurements at 0 mm vertical offset to compare with the measurements at −11 mm vertical offset.

The positioning uncertainty of the detectors in the minibeam field also impacted these correction factors, as their calculation was dependent on the position of the sensitive regions. An estimate of the Type B uncertainty was made by assuming the detectors could be positioned both vertically and horizontally in the minibeam field to within ±1 mm 95% of the time, according to a Gaussian distribution. The trends of the DAP per unit area (similar to those shown in Figure 3, but without normalization) were sampled according to this Gaussian distribution in both the horizontal and vertical directions. The resulting standard deviations of the sampled values were taken as the Type B uncertainties on the DAP per unit area due to horizontal and vertical positioning uncertainties. These standard deviations were added in quadrature for an overall Type B uncertainty estimate due to positioning in 2D. The resultant uncertainties were propagated through the calculation of the $k_{vert}$ and $k_{prof}$ correction factors, and are shown in Table 2.

The Type B uncertainties are significantly reduced for the SOBP for the reasons discussed in Section 3.1, as these correction factors are dependent on the position of the sensitive regions.

### 3.5. NPL PSPC Measurements

**Figure 7:**
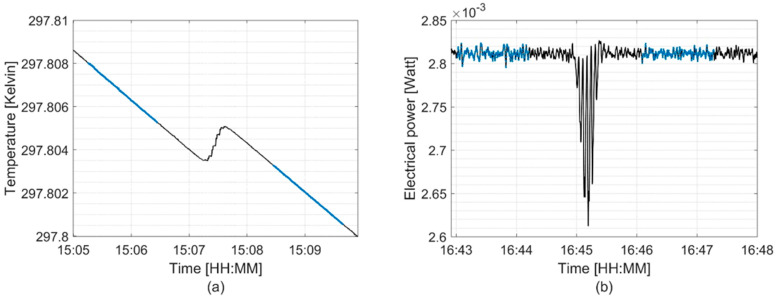
Signal recorded in the calorimeter core during operation in (**a**) quasi-adiabatic and (**b**) active isothermal modes; obtained at 2.0 cm WED using a 100 MeV mono-energetic pMBRT field. Blue data points indicate the pre-irradiation and post-irradiation drifts used for analysis.

**Figure 8:**
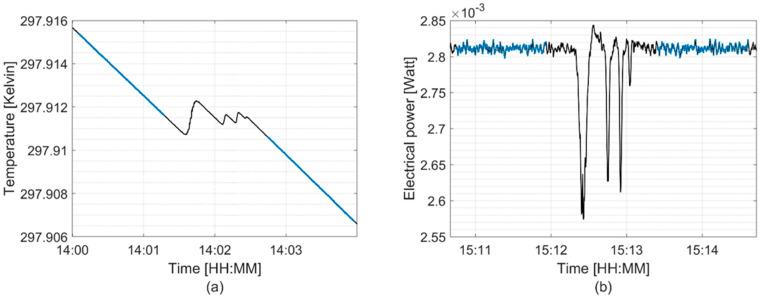
Signal recorded in the calorimeter core during operation in (**a**) quasi-adiabatic and (**b**) active isothermal modes; obtained in the middle (10.0 cm WED) of a 2 cm wide pMBRT SOBP. Blue data points indicate the pre-irradiation and post-irradiation drifts used for analysis.

Figure 7 and Figure 8 show examples of NPL PSPC measurements for the 100 MeV mono-energetic proton field at 2.0 cm WED and the SOBP at 10.0 cm WED, respectively, in both quasi-adiabatic and active isothermal modes. The distinct steps observed in Figure 8 are a result of the switching between energy layers to build the SOBP.

**Figure 9:**
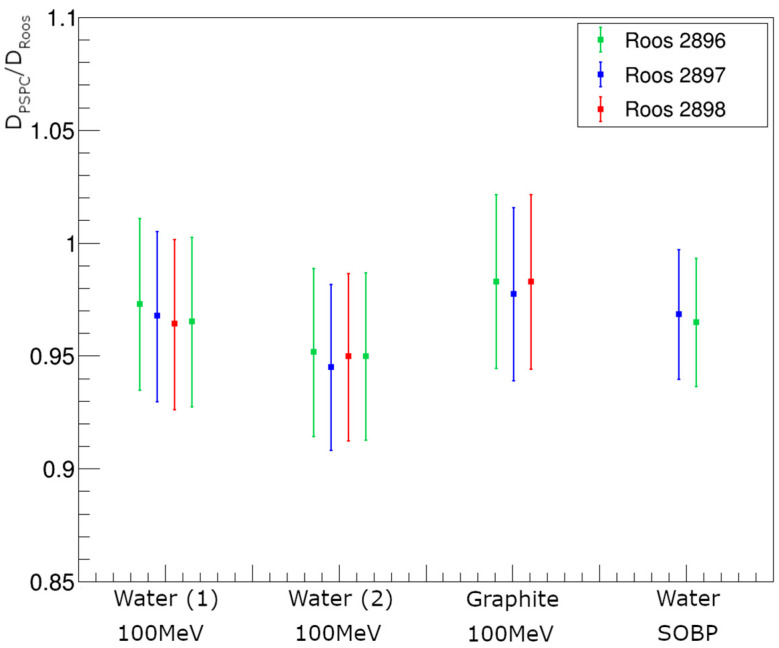
Ratio of dose-to-water measured with the NPL PSPC relative to that measured with three PTW Roos chambers at the coordinates (0, −11) mm from the minibeam field center. Water (1) and Water (2) refer to independent set-ups of the water phantom for PTW Roos chamber measurements. Graphite refers to the buildup for the PTW Roos being made of graphite, and Water SOBP refers to measurements in the SOBP field.

Figure 9 shows the dose-to-water measured with the NPL PSPC relative to that measured with the PTW Roos chambers at a position of (0, −11) mm, relative to the center of the field. Measurements are grouped according to their experimental set-up, which highlights the potential for systematic differences between measurement sets due to the positioning. In Figure 9, Water (1) 100 MeV and Water (2) 100 MeV refer to different sets of measurements following the independent set-up of the water phantom with PTW Roos chambers in the 100 MeV mono-energetic field. Graphite 100 MeV refers to PTW Roos measurements behind graphite buildup plates to achieve the same WED in the 100 MeV mono-energetic field. Water SOBP refers to measurements of the PTW Roos chambers in a water phantom with the SOBP field. The uncertainties shown include all Type A and Type B contributions to the measurements.

## 4. Discussion

The uncertainty regarding the positioning of the detectors in the minibeam field has been shown to be the most influential factor in the uncertainty of the measured dose. It was demonstrated that a 2 mm horizontal offset to the NPL PSPC or PTW Roos chamber, which are of similar size, increased the dose by approximately 8% in the 100 MeV mono-energetic minibeam field described. Uncertainties of 1.4% and 1.1% were attributed to the uncertainty of the dose measured by the NPL PSPC and the PTW Roos chambers, respectively, originating purely from the positioning of the detectors in this field. The significance of these uncertainties is highlighted by the fact that the combined relative standard uncertainty for the determination of absorbed dose-to-water in homogeneous mono-energetic and SOBP fields as recommended in IAEA TRS-398 (Rev. 1, updated in 2024) is 1.7%, which considers uncertainties from all sources. It is therefore critical to find techniques suitable to target the reduction in the uncertainty associated with the position of the detectors in minibeam fields.

The same effect was observed for the SOBP field, but to a lesser extent. A difference in the dose of approximately 3% was observed at a 2 mm horizontal offset compared with central alignment in the SOBP field. Uncertainties of 0.4% and 0.4% were attributed to the uncertainty of the dose measured by the NPL PSPC and PTW Roos chamber, respectively, purely due to the achievable detector positioning accuracy in this field. The reduced uncertainty compared with the mono-energetic field was due to the increased scatter of the protons at the larger 10.0 cm WED of measurement compared with 2.0 cm WED. This increased scatter caused a reduction in the peak-to-valley dose ratio, meaning that any horizontal position offset had less of an impact on the measured dose.

Positioning uncertainties in depth would also contribute to the overall uncertainty, but have not been considered in detail here. Any change in depth would change the dose distribution integrated over the sensitive area of the detector, due to the differing proton scatter. The experimental measurements performed did not facilitate analysis of this like that performed in the horizontal and vertical directions. Estimating the contribution to the uncertainty due to the positioning uncertainty in depth would be possible through Monte Carlo-based modelling of the beam, but this would require a well optimized model of the experimental beam.

The investigation of the influence of the positioning uncertainty due to different sized detectors, despite some not being used in the experimental campaign, has been valuable in highlighting techniques for improvement. The PTW Advanced Markus was shown to have an 8% reduction in the dose measurement with the same 2 mm horizontal offset considered for the PTW Roos and NPL PSPC. However, consideration of the much larger PTW Bragg Peak chamber demonstrated a <1% reduction in the dose measured for the same offset. This significantly reduced difference demonstrated that a larger detector which encapsulates more of the beam may offer reduced uncertainty on the measured dose, as it is less susceptible to the difference in measured dose resulting from encapsulating additional or fewer beamlets.

The attempt made to optimize the tilt of the collimator to achieve a more experimentally representative field in the simulations was unsuccessful. However, this emphasized the difficulties with designing precise and reproducible experimental set-ups of such fields, as well as important considerations required for evaluation of dosimetric uncertainties. It should not be assumed that minibeam fields are easily reproducible when using collimators. Fluctuations in the spatial and angular distribution of the incident spot-scanned beam on the collimator, as well as the tilt of the collimator itself, can lead to significant deviations in the produced field. The difficulties optimizing the field in the simulation indicate that additional quality assurance will likely be required for these fields, due to their spatial complexity. This uncertainty associated with the tilt of the collimator was the predominant contribution to the uncertainty of the Monte Carlo-derived $k_{imp}$ and $k_{gap}$ correction factors.

The film-derived $k_{vert}$ correction factor was required to correct the measurements made in the center of the field to compare with the measurements made at a −11 mm vertical displacement. The inhomogeneity along individual beamlets led to a difference of 3.6% and 2.3% in the dose measured between the center of the field and that with the vertical displacement for the 100 MeV mono-energetic and SOBP fields, respectively. Their respective uncertainties, considering Type A and Type B, of 1.6% and 0.5%, were predominantly impacted by the position uncertainty of the detectors, as the correction is dependent on the location of the sampled region. The magnitude of these corrections emphasizes the need to ensure that the beam is optimized fully in the experiment, to achieve uniformity along individual beamlets. No correction factor would be required with uniformity along the beamlets, which reduces the uncertainty on the dose. Non-uniformity along beamlets would also likely impact and complicate the desired outcome of experiments, as the peak-to-valley dose ratio would be non-uniform over the field area. The significantly reduced uncertainty in the $k_{vert}$ correction factor for the SOBP compared with the mono-energetic beam is due to the increased scatter of the protons at the larger depth of 10.0 cm WED compared with 2.0 cm WED. This scatter makes the dose distribution more uniform, which leads to the uncertainty in the position of the sensitive regions having less impact on the measured dose.

The ratios between the dose-to-water measured using the NPL PSPC compared to that with the PTW Roos chambers shown in Figure 9 demonstrate large differences, in some cases larger than 5%. There is, however, a degree of consistency between the measurements within individual measurement sets. The consistency observed within measurement sets, alongside the larger systematic difference between sets, suggests that the dominant factor impacting the results is the set-up of the phantom and calorimeter positions. Mounting different detectors in an already positioned phantom is more reproducible than repositioning the phantom after it has been removed from the beam. This highlights the importance of developing reproducible experimental set-ups for dosimetry measurements.

One technique to reduce the uncertainty due to positioning is demonstrated in Section 3.1. The larger PTW Bragg peak chamber which encapsulated the majority of the beam demonstrated a significantly reduced uncertainty of the dose. This can be extended further by using a device which encapsulates the entire beam and instead measures Dose-Area Product. Such a technique would have little-to-no dependence on the horizontal position of the detector. This solution would remove the contributions to Type B uncertainties based on the positioning of the detector, as well as removing the requirement for a film measurement.

An alternative solution is to use a device suitable for accurately measuring the beam position and distribution across the calorimeter core. A silicon pixel device has been previously considered [58,59,60,61], and is a likely candidate for application in the minibeam modality. This device could be positioned in front of the calorimeter and the pixels accurately assigned to a position on the calorimeter core. The resulting distribution observed with the pixel device during irradiation could then be used to identify the sampled area of the field with the NPL PSPC. The dose measured in the core could then be corrected for any difference from the target position in the minibeam field, using film dosimetry. Further work is required to address complexities such as charge sharing and the impact of radiation damage before the dose could be corrected from the pixel device without the need of film. It would be possible, through this approach, to significantly reduce the impact of the positioning uncertainty on the dose measurement by utilizing the measurement of any offset present. However, the introduction of an ancillary detector in front of the NPL PSPC would produce additional scatter and consequently impact the measured dose. Also, this approach does still require the utilization of film dosimetry for determination of a positional correction factor, which introduces uncertainty, unlike the DAP approach. An alternative approach potentially overcoming both drawbacks, is to position a film behind the detectors, aiming, by a careful selection of beam energy, to visualize both the beam profile and the contours of the detector components.

## 5. Conclusions

The spatial fractionation involved in proton minibeam radiation therapy makes the dosimetry measurements incredibly challenging. This study has been critical for understanding the current status of performing dosimetry with a primary standard level device as a proof-of-concept, and identifying the necessary improvements.

The precision of the collimator engineering, collimator mounting to the nozzle, and beam alignment with the collimator, have been highlighted here as being of critical importance for the reduction of uncertainties with regard to the dosimetry. More precise design and alignment of the components would have likely reduced the uncertainty originating from the apparent tilt of the collimator, and therefore reduced the uncertainty of the dosimetry. This emphasizes the care that should be taken to ensure that appropriately designed and robust components and beam settings are used for the production of these beams.

The positioning uncertainty has been shown to be the dominant contribution to the uncertainty of the dose measurements, and impacts both experimental measurements and correction factor calculations. Consideration of the impact of using different sized detectors for the dose measurement highlighted the fact that a larger area detector significantly reduced the uncertainty. This approach measures the DAP, and a calorimeter measuring this quantity has been under development at NPL. Using such an approach for the calorimetry may remove or significantly reduce the 1.4% and 0.4% Type B uncertainties of the raw dose measurement associated with the positioning in a 100 MeV mono-energetic and SOBP field, respectively. It would also remove the contribution to the uncertainty of the $k_{vert}$ and $k_{prof}$ correction factors, resulting in a level of uncertainty comparable to that for the conventional proton modality. The significant reduction in the uncertainty of the dose measurement in the SOBP in comparison to the mono-energetic beam also suggests that an approach to the dosimetry which utilizes measurement within a SOBP region could be a more accurate technique to perform the dosimetry in spatially fractionated fields.

Whilst still preclinical, the accurate dosimetry that these improvements will deliver will play a vital role in helping to identify the dose requirements to trigger the beneficial effects observed in SFRT, and the dose limits to alleviate the negative side effects. Once established, the improved accuracy of the primary standard dosimetry will play a key role in maintaining traceability and safe implementation of this novel modality in the clinic.

## Figures and Tables

**Table 1:** Monte Carlo-derived correction factors for a 100 MeV mono-energetic and SOBP pMBRT field. All uncertainties quoted are k = 1.

Correction Factor	100 MeV Mono-Energetic	Uncertainty		SOBP	Uncertainty	
		Type A	Type B		Type A	Type B
$k_{imp}$	1.0013	0.06%	0.08%	1.0012	0.05%	0.10%
$k_{gap}$	1.0002	0.06%	0.11%	0.9984	0.05%	0.24%
$D_{w}^{MC}/D_{g}^{MC}$	1.1164	0.05%	0.48%	1.1428	0.09%	1.26%

**Table 2:** Film-derived correction factors for a 100 MeV mono-energetic and SOBP pMBRT field. All uncertainties quoted are k = 1.

Correction Factor	100 MeV Mono-Energetic	Uncertainty		SOBP	Uncertainty	
		Type A	Type B		Type A	Type B
$k_{vert}(Roos)$	0.9640	0.37%	1.55%	0.9769	0.27%	0.42%
$k_{vert}(PSPC)$				0.9766	0.25%	0.45%
$k_{prof}$	0.9949	0.01%	1.75%	0.9988	0.02%	0.51%

## Data Availability

The data and models generated during the study are available from the corresponding author upon reasonable request. Researchers interested in these resources for further study or replication of our work are encouraged to contact the corresponding author.
